# High Frequency of CD8 Positive Lymphocyte Infiltration Correlates with Lack of Lymph Node Involvement in Early Rectal Cancer

**DOI:** 10.1155/2014/792183

**Published:** 2014-12-30

**Authors:** Silvio Däster, Serenella Eppenberger-Castori, Christian Hirt, Inti Zlobec, Tarik Delko, Christian A. Nebiker, Savas D. Soysal, Francesca Amicarella, Giandomenica Iezzi, Giuseppe Sconocchia, Michael Heberer, Alessandro Lugli, Giulio C. Spagnoli, Christoph Kettelhack, Luigi Terracciano, Daniel Oertli, Urs von Holzen, Luigi Tornillo, Raoul A. Droeser

**Affiliations:** ^1^Department of Surgery, University Hospital Basel, Spitalstrasse 21, 4031 Basel, Switzerland; ^2^Institute of Surgical Research and Hospital Management (ICFS) and Department of Biomedicine, University of Basel, Hebelstrasse 20, 4031 Basel, Switzerland; ^3^Institute of Pathology, University Hospital Basel, Schoenbeinstrasse 40, 4031 Basel, Switzerland; ^4^Institute of Pathology, University of Bern, Murtenstrasse 31, 3010 Bern, Switzerland; ^5^Institute of Translational Pharmacology, Department of Biomedicine, National Research Council, Via Fosso del Cavaliere 100, 00133 Rome, Italy

## Abstract

*Aims*. A trend towards local excision of early rectal cancers has prompted us to investigate if immunoprofiling might help in predicting lymph node involvement in this subgroup.* Methods*. A tissue microarray of 126 biopsies of early rectal cancer (T1 and T2) was stained for several immunomarkers of the innate and the adaptive immune response. Patients' survival and nodal status were analyzed and correlated with infiltration of the different immune cells.* Results*. Of all tested markers, only CD8 (*P* = 0.005) and TIA-1 (*P* = 0.05) were significantly more frequently detectable in early rectal cancer biopsies of node negative as compared to node positive patients. Although these two immunomarkers did not display prognostic effect “per se,” CD8+ and, marginally, TIA-1 T cell infiltration could predict nodal involvement in univariate logistic regression analysis (OR 0.994; 95% CI 0.992–0.996; *P* = 0.009 and OR 0.988; 95% CI 0.984–0.994; *P* = 0.05, resp.). An algorithm significantly predicting the nodal status in early rectal cancer based on CD8 together with vascular invasion and tumor border configuration could be calculated (*P* < 0.00001).* Conclusion*. Our data indicate that in early rectal cancers absence of CD8+ T-cell infiltration helps in predicting patients' nodal involvement.

## 1. Introduction

Colorectal cancer (CRC) is the third most common cause of cancer-related death in both men and women worldwide [1]. Approximately 40% of all CRCs are located in the rectum [2]. The standard treatment of stage I rectal cancer (RC) is rectal resection with total mesorectal excision (TME). In locally advanced disease (stages II and III), management consists of neoadjuvant chemoradiotherapy (CRT) followed by TME [3]. However, in early rectal cancer (ERC), local excision is often considered as curative treatment alternative to TME. Transanal endoscopic microsurgery (TEM) is an accepted first-line therapy for T1 low-risk RCs [4]. Recent data have indicated the potential to also treat nodal-negative T2 and T3 tumors in combination with preoperative CRT with local excision [5–8]. However, one of the limitations of local excision is represented by the impossibility to determine the pN-category. Lymph node involvement in RC is known to correlate with T stage. 10–15% of T1 RCs are associated with nodal positivity, whereas in T2 tumors the risk for nodal metastases is 19% [9] and rises to >50% in T3 and T4 tumors [5, 10].

There is increasing evidence that immunoprofiling might help to predict clinical outcome in CRC, possibly more reliably than TNM classification or grading [11–14]. For instance, loss of E-cadherin has been shown to independently predict lymph node involvement in a cohort of 221 CRCs [15], and, in general, there is evidence that a low number of tumor-infiltrating lymphocytes (TILs) predict lymph node involvement in melanoma [16], gastric cancer [17], breast cancer [18], and cervical cancer [19].

Until now, it has not been possible to identify a correlation between tumor infiltration by immune cells and lymph node involvement in rectal cancer. The primary objective of this study was to determine if there are immunohistochemical markers associated with lymph node positivity in ERC (T1 and T2). To do this, we investigated a tissue microarray (TMA) of 126 biopsies of ERC patients, stained for several markers of the innate and adaptive immune response. We analyzed the possibility to predict the histopathological nodal state by building an algorithm based on the infiltration by cells expressing different immunomarkers.

## 2. Materials and Methods

### 2.1. Tissue Microarray Construction

The cohort consisted of nonconsecutive, primary ERCs (*n* = 126). The technique of TMA construction has previously been described [20]. Briefly, tissue cylinders of formalin-fixed, paraffin-embedded ERC tissue blocks with a diameter of 0.6 mm were obtained, punched from morphologically representative areas of each donor block, and brought into one recipient paraffin block (30 × 25 mm), using a semiautomated tissue arrayer. Each punch was made from the center of a tumor so that each TMA spot consisted of at least 50% tumor cells. All biopsies were collected within the activities of the biobank of the Institute of Pathology, University Hospital Basel, and the study was approved by the local ethical committee (EKBB).

### 2.2. Clinicopathological Features

Clinicopathological data for the 126 ERC patients included in the TMA were collected retrospectively in a nonstratified and nonmatched manner. Annotation included patient age, tumor diameter, location, pN stage, grade (G1–G3), histological subtype, presence of vascular invasion, tumor border configuration (infiltrative or pushing), and disease-specific survival (Table 1). Tumor border configuration was evaluated according to Jass using the original H&E slides of the resection specimens corresponding to each tissue microarray punch [21]. In addition, the microsatellite status of the tumors was established [22]. Overall survival was defined as primary endpoint. Follow-up data were available for 123 patients with mean/median event-free follow-up time of 74/75 months.

### 2.3. Immunohistochemistry

Standard indirect immunoperoxidase procedures were used for immunohistochemistry (IHC; ABC-Elite, Vector Laboratories, Burlingame, CA). Briefly, slides were dewaxed and rehydrated in distilled water. Endogenous peroxidase activity was blocked using 0.5% H_2_O_2_. Sections were incubated with 10% normal goat serum (DakoCytomation, Carpinteria, CA) for 20 min and incubated with primary antibody at room temperature. Primary antibodies used were specific for MPO (clone 59A5, Novocastra, Newcastle, UK), CD16 (clone 2H7, Novocastra), CD56 (Dako, Glostrup, Denmark), CD68 (clone PG-M1, Dako, Glostrup, Denmark), CD163 (monoclonal, NeoMarkers, MS-1103), CD3 (monoclonal, Dako, Glostrup, Denmark), CD4 (monoclonal, NeoMarkers, MS-1528), CD8 (clone C8/144B, DakoCytomation, Switzerland), CD45RO (Thermo Scientific UCHL-1, monoclonal), TIA-1 (monoclonal, Immunotech, IM2550), FOXP3 (clone 236A/E7, Abcam, Cambridge, UK), and IL-17 (purified polyclonal goat anti-human IL-17 antibody, R&D Systems, Minneapolis, USA; polyclonal rabbit IL-17 antibody sc-7927, H-132, Santa Cruz Biotechnology, Santa Cruz, USA). As primary PD-L1 (CD274) specific reagent, a monoclonal antibody was used (mAb, clone 27A2, MBL, Woburn, MA, USA) [23]. The HLA-DR, DQ, DP antigen mAb, LGII-612.14 was developed and characterized as described [24, 25]. Subsequently, sections were incubated with peroxidase-labeled secondary antibody (DakoCytomation) for 30 min at room temperature. For visualization of the antigen, sections were immersed in 3-amino-9-ethylcarbazole plus substrate-chromogen (DakoCytomation) for 30 min and counterstained with Gill's hematoxylin.

### 2.4. Evaluation of Immunohistochemistry

Tumor infiltrating cells positive for the abovementioned immune markers were counted for each punch (approximately one high power (20x) field) by two trained research fellows (Francesca Amicarella and Raoul A. Droeser). Data were independently validated by an additional investigator (Luigi Tornillo). Representative pictures of tumor samples stained with different immunomarkers are illustrated in a previously published study from our group [26].

### 2.5. Statistical Analysis

Cut-off scores used to classify ERCs with low or high infiltration were obtained by regression tree analysis, evaluating sensitivity and false positive rate for the discrimination of survivors and nonsurvivors, on all tumor samples [27]. Wilcoxon test was used to determine the association of markers of immune cell infiltration and the nodal status. Survival analysis was carried out by Cox regression analysis and Kaplan-Meier curves were compared by log rank test. Predictive value of immunomarker expression with respect to nodal status was calculated by logistic regression.

Subsequently, immune markers data and clinicopathological features that were found to be significantly associated with lymph node involvement in univariate analysis were entered into a multivariate logistic model. The regression coefficients were estimated using maximum likelihood estimation. Statistical analyses were performed using R (Version 2.15.2, http://www.r-project.org/).

## 3. Results

### 3.1. Clinicopathological Characteristics of the Patients Cohort

The patients cohort consisted of 126 ERCs. The mean age at diagnosis was 68 years (median age 69; range 36–91). According to the TNM classification, there were 24 (19%) pT1 and 102 (81%) pT2 tumors. Furthermore there were 97 (77%) pN0, 22 (17.5%) pN1, and 7 (5.5%) pN2 cases. Tumor grade was G1 in 6 (4.7%) cases, G2 in 116 (92%) cases, and G3 in 4 (3.3%) cases. In 50 (40%) biopsies, an infiltrative tumor border configuration was observed and in the majority of cases vascular invasion was absent (*n* = 111, 88%). Finally, most of the tumors belonged to the microsatellite stable subgroup (*n* = 119, 94.4%) and 5-year survival rate was 79.2% (95% CI 72.2–86.9). The clinicopathological characteristics of the patients cohort are summarized in Table 1. Due to the low number of cases with pN2 (≥4 positive lymph nodes), the overall analysis was performed combining pN2 together with the pN1 cases versus pN0. The pN status was determined by analyzing on average 9.6 lymph nodes (range 1–61).

### 3.2. Validation of Patients Cohort

To verify whether our patients cohort was representative, we performed a survival analysis according to the nodal involvement. As expected, in our cohort of ERCs, nodal positive patients had significantly worse survival as compared to nodal negative patients (*P* = 0.0001; supplementary Figure 1) (see Supplementary Material available online at http://dx.doi.org/10.1155/2014/792183). Furthermore, the nodal positive group included a significantly higher number of patients with an infiltrative tumor border configuration (*P* = 0.03; supplementary Figure 2) and vascular invasion (*P* = 0.01; supplementary Figure 3). Since most of the cases were of grade 2 (92%) and the vast majority of tumors (94.4%) were microsatellite stable, we did not perform a survival analysis for these factors.

### 3.3. Distribution of Innate Immune Markers according to Nodal Involvement in ERC

To explore relationships occurring between tumor infiltration by cells of the innate immune system and lymph node involvement, distribution of MPO, CD16, CD56, CD68, and CD163 positive cells was compared in the nodal negative and nodal positive cancers. However, no significant differences were observed (Table 2(a)).

### 3.4. Distribution of Adaptive Immune Markers according to Nodal Involvement in ERC

**Figure 1:**
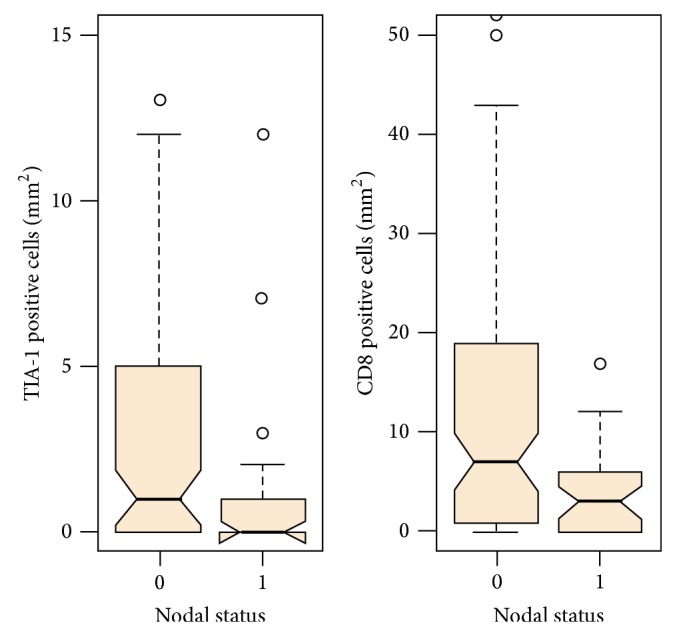
Boxplot distribution of CD8 (*P* = 0.005) and TIA-1 (*P* = 0.05) positive T-cell infiltration per mm^2^ according to nodal involvement in a cohort of early rectal cancers (*n* = 126).

To explore relationships occurring between tumor infiltration by cells of the adaptive immune system and lymph node involvement, distribution of T-cell markers such as CD3, CD4, CD8, CD45RO, PD-L1, HLA class II, TIA-1, FoxP3, and IL-17 was comparatively evaluated in nodal negative and nodal positive tumors. Of all markers tested, only CD8 (*P* = 0.005) and TIA-1 (*P* = 0.05) were found to be significantly more frequently detectable in ERC biopsies of nodal negative as compared to nodal positive patients (Table 2(b); Figure 1).

### 3.5. Prognostic and Predictive Significance of CD8 and TIA-1 Cell Infiltration in ERC

Neither CD8 nor TIA-1 displayed any prognostic effect “per se” (*P* = 0.76 and *P* = 0.09, resp.). However, if their values were entered into a univariate logistic regression, CD8 resulted to be significantly predictive for the nodal involvement (OR 0.994; 95% CI 0.992–0.996; *P* = 0.009). Regarding TIA-1, only a trend could be observed in the logistic regression analysis for prediction of nodal involvement (OR 0.988; 95% CI 0.984–0.994; *P* = 0.05, Table 3).

Furthermore, by using the published threshold of 10 for CD8 [26], the OR was 0.258 (CI 0.145–0.459; *P* = 0.019) in univariate analysis. The TIA-1 cut-off for survival evaluation was 0 and 1 cell per mm^2^. With this threshold, the logistic regression predicting the nodal status was not significant (OR 0.406; CI: 0.225–0.731; *P* = 0.125).

### 3.6. Validation in a Cohort of Early Stage Colon Cancers

To evaluate if our predictions could be transferred to another independent patients cohort, we performed the distribution (Wilcoxon) and logistic regression analysis in an independent cohort of early stage colon cancers (*n* = 114). In this cohort, a similar fraction of nodal positive patients (*n* = 26, 22.8%) was present. As expected, nodal negative patients had significantly better survival compared to the nodal positive group in this cohort (*P* < 0.00001; HR 3.57; CI 3.25–3.89). For TIA-1, we could confirm the asymmetric distribution that was detectable in the ERCs cohort with a higher number of cells in nodal negative biopsies (*P* = 0.02). However, differential distribution of CD8+ lymphocytes between the nodal negative and nodal positive group only showed a trend towards a higher infiltration in nodal negative biopsies (*P* = 0.098). Finally, the results of univariate logistic regression analysis did not display any significance for CD8 (*P* = 0.31), TIA-1 (*P* = 0.13), or any other immune markers. However, vascular invasion (OR 1.47; 95% CI 1.32–1.65; *P* = 0.0005) and tumor border configuration (OR 1.23; 95% CI 1.13–1.33; *P* = 0.01) retained a predictive value in this comparative cohort.

### 3.7. Multivariate Prediction of Lymph Node Involvement

The multivariate logistic regression of the ERCs cohort revealed that CD8 (OR 0.996; 95% CI 0.994–0.998; *P* = 0.025) adds predictive information for lymph node involvement independently of tumor vascular invasion (OR 1.465; 95% CI 1.319–1.628; *P* = 0.0005) and tumor border configuration (OR 1.254; 95% CI 1.169–1.344; *P* = 0.001, Table 3). However, in the multivariate logistic regression analysis of the comparative cohort, the application of the same algorithm for prediction of lymph node involvement was only marginally significant and only due to the impact of vascular invasion (CD8: *P* = 0.61; vascular invasion: *P* = 0.02; tumor border configuration: *P* = 0.166).

Furthermore, in the dichotomized analysis, CD8 showed only a trend for the prediction of lymph node involvement (OR 0.322; CI 0.175–0.592; *P* = 0.063) in the multivariate model.

## 4. Discussion

Our results show that high frequency of CD8+ lymphocyte infiltration is significantly predictive of lack of lymph node involvement in T1 and T2 rectal cancers. We could not demonstrate a significant association for other markers of the adaptive immune system, although activated T-cells expressing the TIA-1 marker correlated with lack of lymph node metastases in a univariate analysis. The infiltration by cells expressing markers usually detectable in macrophages, granulocytes, or natural killer cells did not appear to be associated with nodal involvement.

Cytotoxic T-cells have been demonstrated to be key players in antitumoral immunity [28]. Infiltration by CD8+ cytotoxic T-cells has previously been shown to be strongly associated with favorable clinical outcome in CRC patients in numerous studies and to predict patient survival more efficiently than histopathological staging [11, 12, 26, 29]. The correlation between density of TILs and negative nodal status has already been shown for different types of cancer. Taylor et al. have reported that, in a cohort of 875 patients with melanoma, presence of TILs was independently predictive for absence of sentinel lymph node metastases [16]. Our findings on CD8 positive lymphocyte infiltration associated with lack of lymph node involvement are consistent with those reported by Sheu et al., who showed similar results for breast cancer [18], Lee et al., who demonstrated a correlation between CD8+ TILs and negative lymph node status for gastric cancer [30], and Piersma et al., who showed similar results for early-stage cervical cancer [19].

Previously, some of us could demonstrate that RHAMM and CD8 have an independent prognostic impact in a cohort of 482 rectal cancers [31]. Patients with a negative prognostic combination of the two protein markers and nodal negative status had a comparable survival curve to any nodal positive patient group besides the nodal positive group with high RHAMM and low CD8.

In contrast to other types of cancer, in ERC, the lymph node status is crucial for the choice of surgical therapy, for example, local excision versus TME [32]. Since preoperative lymph node staging with different imaging techniques like CT scan, MRI, PET scan, and endoluminal ultrasound is not sufficient to detect nodal micrometastases, there is a need for additional methods better predicting the probability of lymph node involvement. A recent population-based study has analyzed different risk factors for lymph node metastases in a cohort of 677 T1 and T2 rectal cancers. This study showed T2 stage, poor differentiation, and vascular infiltration to be significant predictors of nodal positivity [33]. Our study confirms and extends these findings: not only high tumor grade and vascular infiltration but also tumor border configuration was significantly associated with lymph node metastases in the multivariate analysis. Other studies have also reported similar results [10, 34, 35].

Our study has several limitations. First, the sample size is relatively small. Therefore, it is possible that other TIL markers did not show a significant correlation with lack of lymph node involvement simply because the study was underpowered. Another limitation of the present study is the TMA method itself, as described elsewhere [36]. Indeed, considering tumor heterogeneity, the small sizes of the TMA punches might lead to sampling errors. Last but not least, it is a retrospective study, where the possibility of selection biases cannot be ruled out. However, while further research is warranted, our data set the stage for a critical analysis of the clinical significance of immune microenvironment in early stage rectal cancers, with obvious consequences for treatment and quality of life of patients.

The patients in this study did not receive neoadjuvant treatment. In a recent study by Lezoche et al., the authors suggested to treat T2 N0 tumors with neoadjuvant chemoradiotherapy followed by local excision [8]. It is questionable whether our results are applicable to that group of patients as well. Furthermore, we only included rectal cancers in this study because of the clinically relevant question. In a separate analysis (data not shown), we also evaluated a cohort of 114 T1 and T2 tumors located in the colon. In this analysis, a similar trend could be observed, although it failed to reach the threshold of statistical significance.

In conclusion, we show for the first time the correlation between high frequency of CD8 positive lymphocyte infiltration and the lack of lymph node involvement in a cohort of ERCs. Our findings suggest that, besides conventional pathological analysis like T stage, tumor size, sm-level of infiltration, differentiation, and vascular infiltration, evaluation of the immunological microenvironment might further improve prediction of lymph node involvement in ERC. The algorithm we calculated has an indicative meaning. The study population is small, but it is clear that absence of CD8 and absence of TIA-1 predict lymph node involvement and in these cases confirmation of the nodal involvement is mandatory. Additional studies are needed to confirm our results. A next step could be represented by the definition of a risk score combining different features of the tumor including TILs and facilitating the identification of cancers where local excision could offer an alternative to TME with an acceptable level of risk.

## Supplementary Material

Supplementary Figure 1 shows the effect of lymph node involvement on overall survival in patients with early rectal cancer, Kaplan-Meier overall survival curves were designed according to nodal positivity. (n =123; 12 deaths observed in 95 patients with no positive lymph node, 10 deaths observed in 21 patients with 1 to 3 positive lymph nodes and 4 deaths observed in 7 patients with more than 3 positive lymph nodes in a cohort of early rectal cancers; P = 0.0001).
Supplementary Figure 2 displays the effect of infiltrative tumor border configuration (P =0.0264) and supplementary Figure 3 the effect of vascular invasion (P =0.00578) on overall survival in patients with early rectal cancer.

## Figures and Tables

**Table 1:** Characteristics of early rectal cancer patients cohort (*n* = 126).

Age, mean, and median (range in years)	68, 69 (36–91)
Tumor size (mm)	36, 35 (12–70)
Gender	
Female	61 (49.6%)
Male	65 (50.4%)
T stage	
T1	24 (19%)
T2	102 (81%)
N stage^*^	
N0	97 (77%)
N1	22 (17.5%)
N2	7 (5.5%)
Tumor grade	
G1	6 (4.7%)
G2	116 (92.0%)
G3	4 (3.3%)
Tumor border configuration	
Infiltrative	50 (39.7%)
Pushing	76 (60.3%)
Vascular invasion	
No	111 (88%)
Yes	16 (12%)
Microsatellite stability	
Proficient	119 (94.4%)
Deficient	7 (5.6%)
Survival mean, median, and range (months)	74, 75, 1–150
5-year survival (95% CI)	79.2% (72.2–86.9%)

^*^Percentages do not add to 100% due to missing values. Survival analysis was performed according to the Kaplan-Meier method.

**(a):** 

Marker	*N* − (n = 97)	*N* + (n = 29)	*P* values^*^
	Median (range)	Median (range)	
MPO	36 (0–126)	34 (1–119)	0.77
CD16	46 (7–100)	36 (1–100)	0.86
CD56	0 (0–55)	1 (0–100)	0.38
CD68	2 (0–3)	2 (0–3)	0.16
CD163	3 (0–3)	2 (1–3)	0.49

^*^According to Wilcoxon test.

**(b):** 

Marker	*N* − (*n* = 97)	*N* + (n = 29)	*P* values^*^
	Median (range)	Median (range)	
CD3	2 (0–3)	2 (1–3)	0.23
CD4	0 (0–45)	0 (0–45)	0.29
CD8	7 (0–125)	3 (0–17)	0.005
CD45RO	2 (0–3)	2 (0–3)	0.97
TIA-1	1 (0–34)	0 (0–12)	0.05
FoxP3	2 (0–3)	2 (0–3)	0.27
IL-17	1 (0–3)	2 (0–3)	0.56
PD-L1	3 (0–3)	3 (1–3)	0.90
HLA-DR	0 (0–100)	0 (0–100)	0.32

^*^According to Wilcoxon test.

**Table 3:** Logistic regression analysis for the prediction of lymph node involvement in ERC.

	Univariate		Multivariate	
	OR (95% CI)	*P* values	OR (95% CI)	*P* values
CD8	0.994 (0.992–0.996)	0.009	0.996 (0.994–0.998)	0.025
TIA-1	0.988 (0.984–0.994)	0.047	—	—
Age	0.998 (0.995–1.00)	0.696	—	—
Gender (m. versus f.)	1.066 (0.989–1.151)	0.391	—	—
pT stage (1, 2)	1.199 (1.090–1.318)	0.058	—	—
Tumor grade (1, 2, 3)	1.415 (1.242–1.612)	0.009	1.434 (1.264–1.627)	0.005
Vascular invasion	1.641 (1.473–1.828)	<0.0001	1.465 (1.319–1.628)	0.0005
Tumor border configuration	1.24 (1.151–1.336)	0.005	1.254 (1.169–1.344)	0.001
Microsatellite stability (deficient versus proficient)	1.276 (1.083–1.502)	0.14	—	—

Uni- and multivariate logistic regression analyses showing OR and *P* values for all ERCs (*n* = 123, less than 126 due to missing values) conferred by CD8 and TIA-1 density, age, sex, tumor size, tumor grade, vascular invasion, tumor border configuration, and microsatellite stability.
