# Supplementary material for: High Frequency of CD8 Positive Lymphocyte Infiltration Correlates with Lack of Lymph Node Involvement in Early Rectal Cancer
Source: Dis Markers. 2014 Dec 30;2014:792183. doi: 10.1155/2014/792183 (PMC4293805; doi:10.1155/2014/792183)
Supplement: Supplementary file 1 — Supplementary Figure 1 shows the effect of lymph node involvement on overall survival in patients with early rectal cancer, Kaplan-Meier overall survival curves were designed according to nodal positivity. (n =123; 12 deaths observed in 95 patients with no positive lymph node, 10 deaths observed in 21 patients with 1 to 3 positive lymph nodes and 4 deaths observed in 7 patients with more than 3 positive lymph nodes in a cohort of early rectal cancers; P = 0.0001). Supplementary Figure 2 displays the effect of infiltrative tumor border configuration (P =0.0264) and supplementary Figure 3 the effect of vascular invasion (P =0.00578) on overall survival in patients with early rectal cancer. [file 792183.f1.pdf]

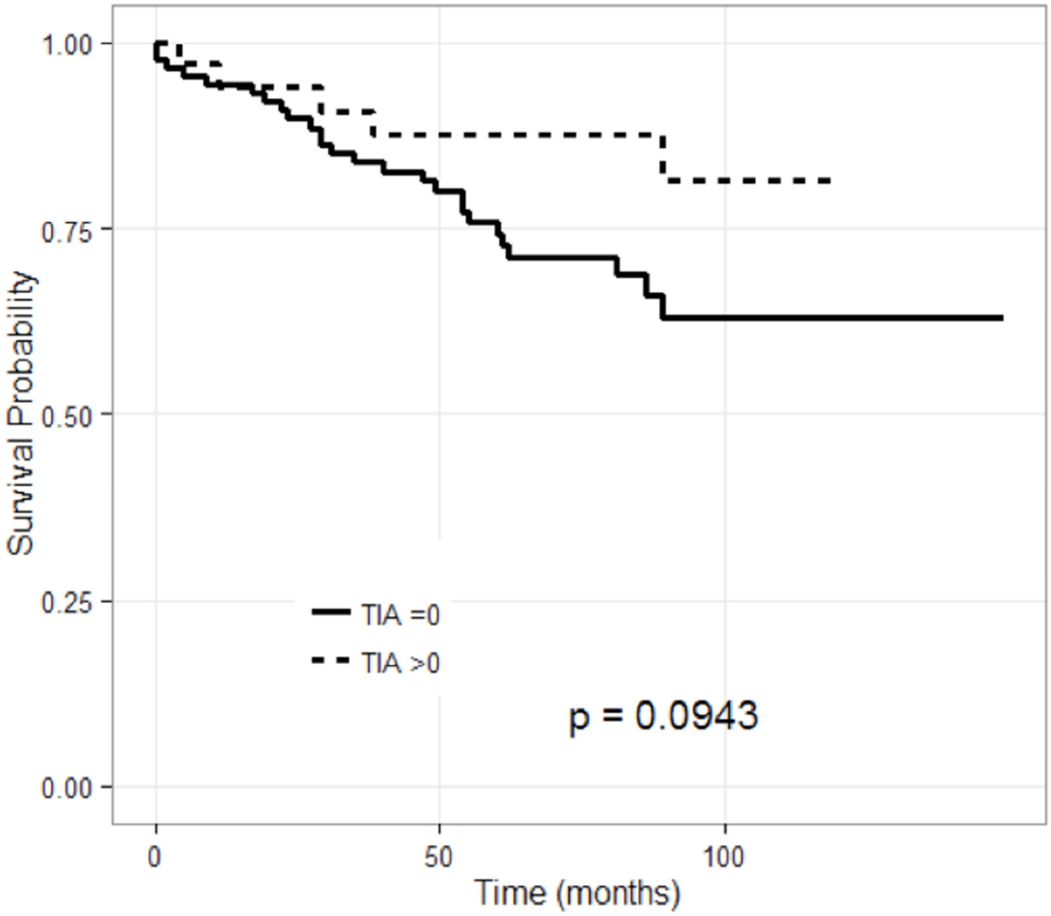

**TIA =0**	94	58	16
**TIA >0**	34	24	8

Numbers at risk

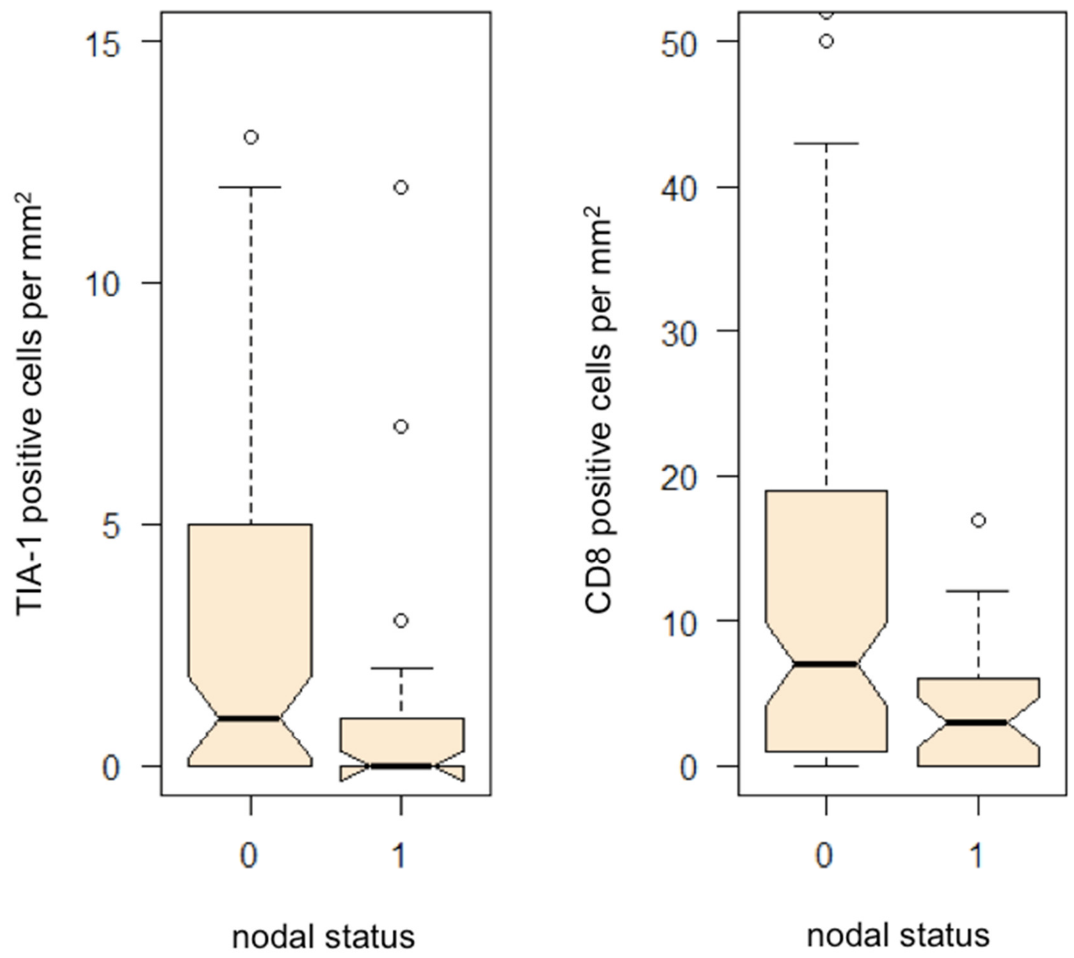

TIA-1 positive cells per mm²
15
10
5
0
0
1
nodal status
CD8 positive cells per mm²
50
40
30
20
10
0
0
1
nodal status

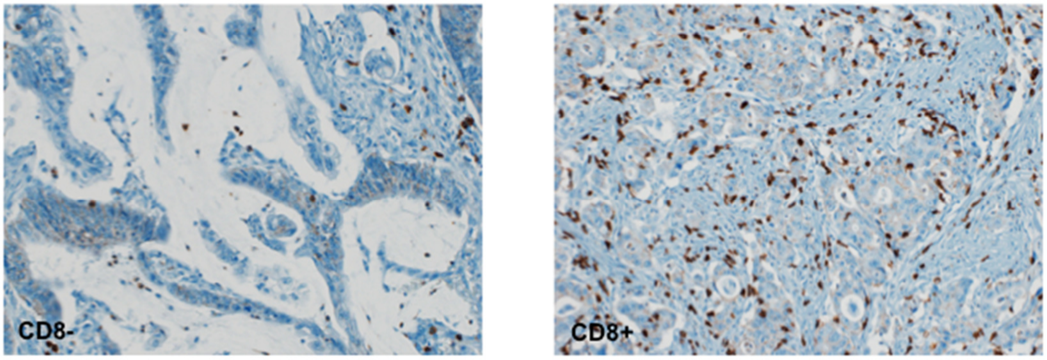

Examples of IHC-staining for CD8: CD8-, less than 10 CD8+ lymphocytes.
CD8+: more than 10 CD8+ lymphocytes.

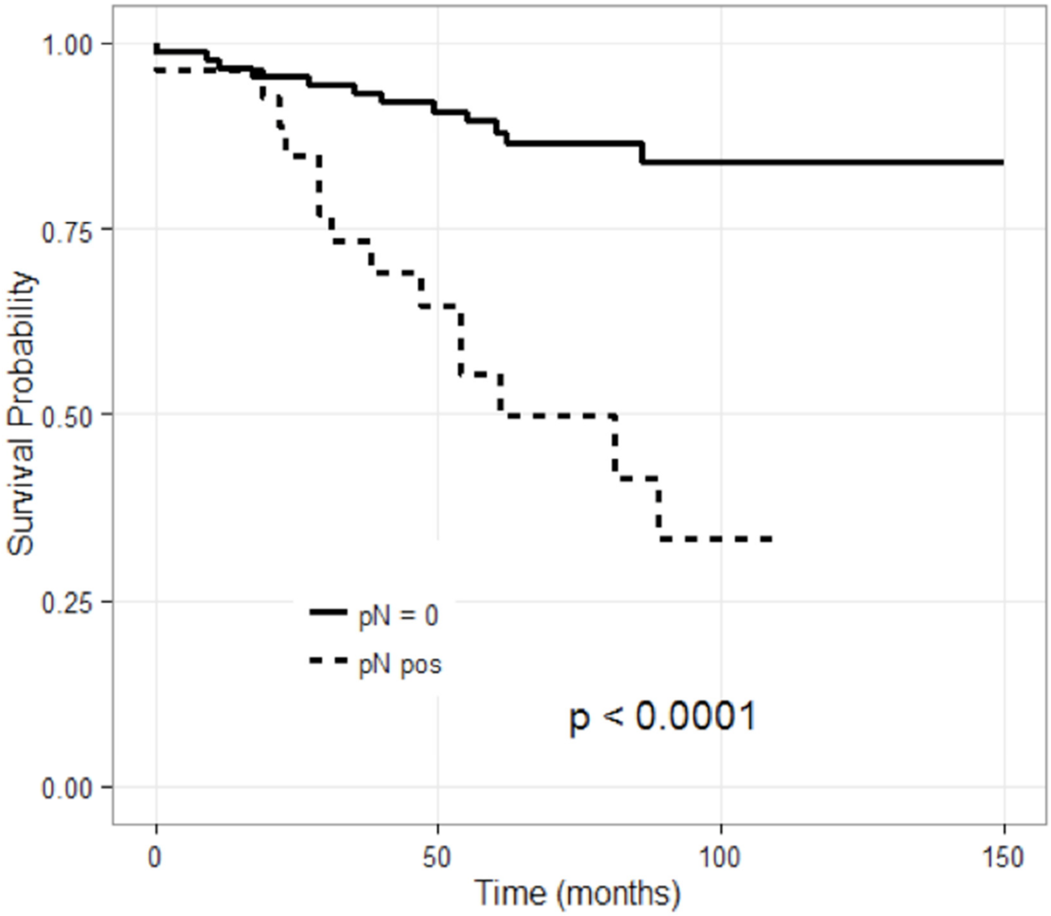

**pN = 0**	95	69	20	1
**pN pos**	28	14	3	0

Numbers at risk

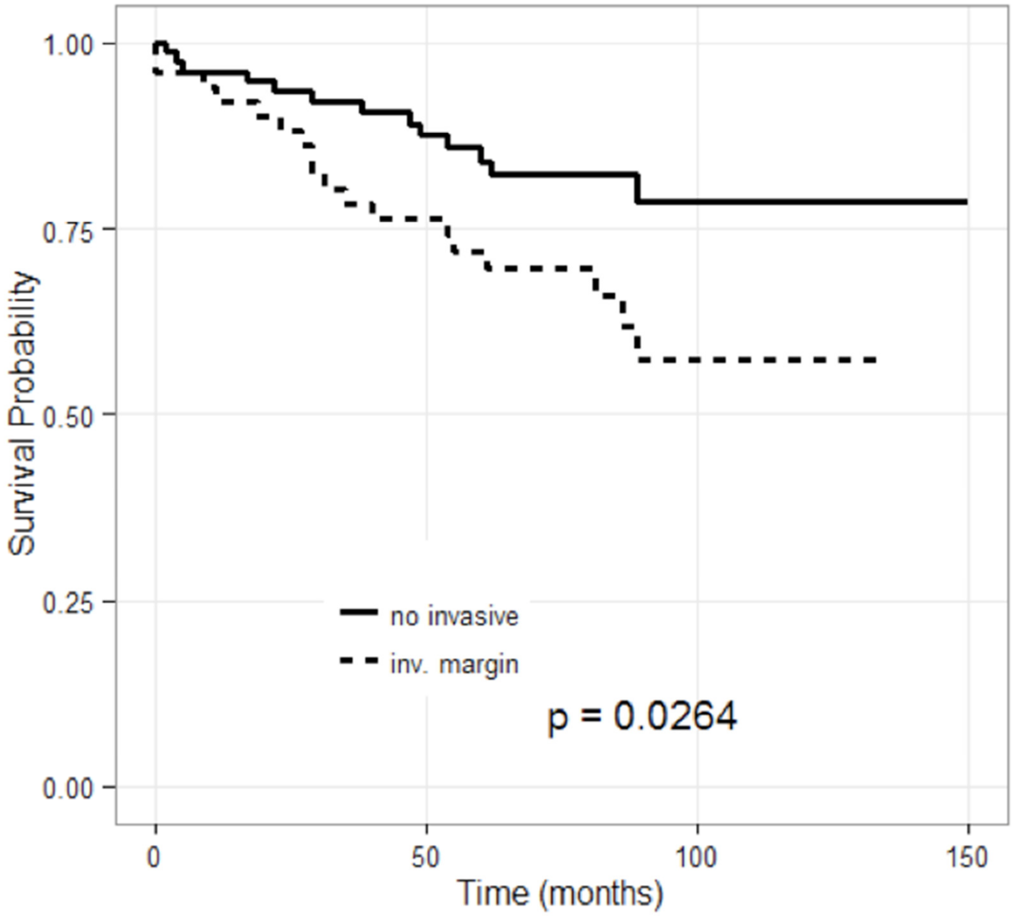

**no invasive**	84	55	16	1
**inv. margin**	52	34	10	0

Numbers at risk

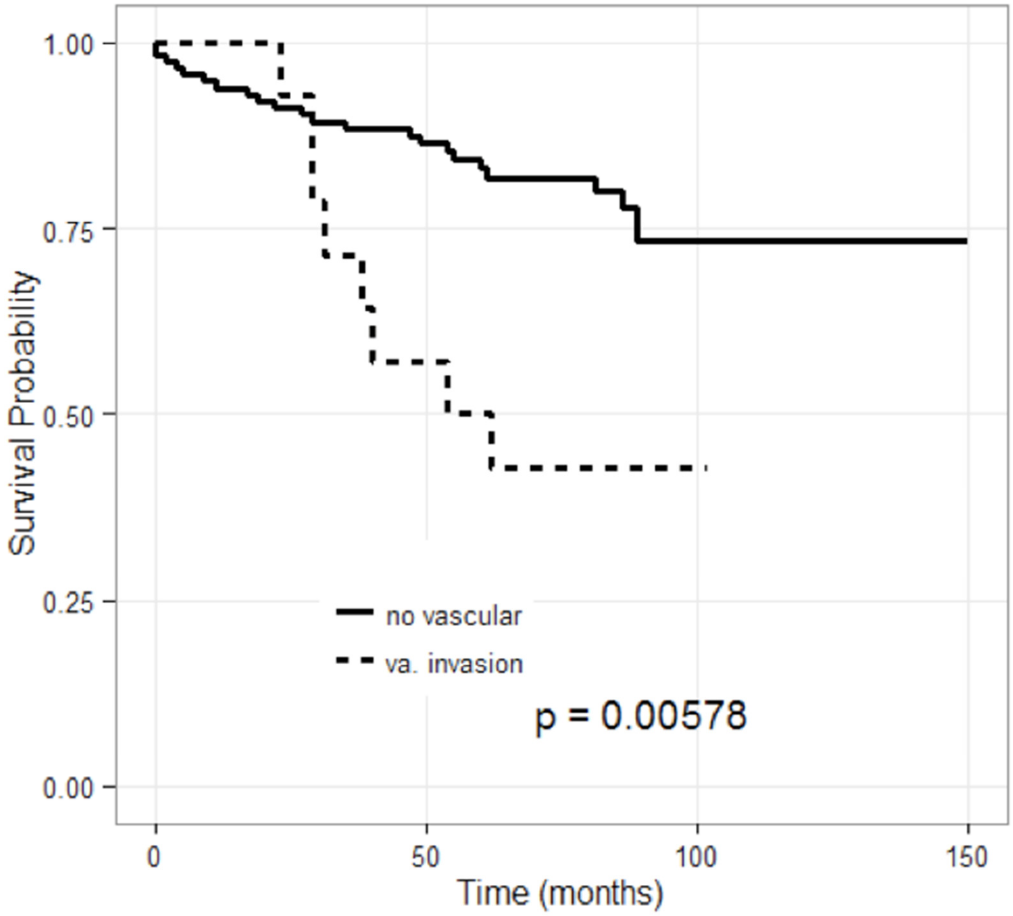

**no vascular**	120	81	23	1
**va. invasion**	16	8	3	0

Numbers at risk
